# Partial Nephrectomy, a Comparison between Different Modalities: A Tertiary Care Center Experience

**DOI:** 10.15586/jkcvhl.v8i2.179

**Published:** 2021-06-17

**Authors:** Ahmed Al Asker, Abdulmalik Addar, Mohammed Alghamdi, Saud Alawad, Mohammed Alharbi, Saeed Bin Hamri, Nasser Albqami, Abdullah Alkhayal, Khaled Alrabeeah

**Affiliations:** 1 Assistant Professor, College of Medicine, King Saud bin Abdulaziz University for Health Sciences, King Abdulaziz Medical City, Division of Urology, King Abdullah International Medical Research Center, Riyadh14611, Saudi Arabia;; 2 Urology Resident, Department of Urology, McGill University, Montréal, QC, Canada;; 3 College of Medicine, King Saud bin Abdulaziz University for Health Sciences, King Abdulaziz Medical City, Division of Urology, King Abdullah International Medical Research Center, Riyadh14611, Saudi Arabia;; 4 Assistant Professer Urology Surgery, College of Medicine, King Saud bin Abdulaziz University for Health Science, King Abdulaziz Medical City, Division of Urology, King Abdullah International Medical Research Center, Riyadh14611, Saudi Arabia

**Keywords:** partial nephrectomy, robotic surgrey, nephron sparing, ischemia time

## Abstract

Kidney cancer, with 4% of all malignancies, is one of the most common malignancies occurring among in adults. In Saudi Arabia, kidney cancer comprises 2.3% of all cancers, and its incidence has increased by 33%. Partial nephrectomy (PN) is considered as the gold standard for T1 renal masses.

In this retrospective study, we did a chart review for all patients who underwent PNs between April 2013 and February 2019. Data comprised presentation, tumor size, type of procedure (open vs. laparoscopic vs. robotic), and intra- and post-operative complications. Chi-square, ANOVA, and cross-tabulation were done using SPSS software. P > 0.05 was considered significant. Approval was obtained from the institutional review board of King Abdullah International Medical Research Center.

In all, 69 patients were identified: 26 (37.7%) males and 43 (62.3%) females, with mean age = 54.53 ± 13.21 years; mean body mass index = 32.36 ± 7.03, and mean tumor size = 3.7 ± 1.72 cm. In terms of presentation, most patients (50, 72.4%) presented incidentally as opposed to symptomatic presentation. Of these patients, 18 (26.1%) underwent *open* partial nephrectomy (OPN), 29 (42%) laparoscopic partial nephrectomy (LPN), and 22 (31.9%) robotic partial nephrectomy (RPN). On comparing minimally invasive surgery (MIS) PN with OPN, we found that OPN had more blood loss and a longer hospital stay but a shorter operating room (OR) time.

Results of PN irrespective of the procedure type, whether it was OPN, LPN, or RPN, were similar if performed by experienced surgeons. However, open procedures involved a higher blood loss, more operative time, and longer hospital stay when compared with minimally invasive techniques.

## Introduction

In managing organ-confined tumors, radical resection has been the preferred management. In renal cell carcinoma (RCC), radical nephrectomy was the gold standard in approaching solid renal masses. However, with the development of minimally invasive surgery (MIS), laparoscopic and robotic approaches have been used for treating RCC (1). Nephron sparing surgery, partial nephrectomy (PN), has emerged in treating solid renal masses of 4–7 cm in diameter or clinical T1a and T1b renal masses. According to current guidelines of the American Urological Association and European Urological Association, PN is the standard of care for small renal masses, with robotic partial nephrectomy (RPN) and laparoscopic partial nephrectomy (LPN) being valid MIS options (2–4). PN has the advantage of preserving renal function, and thus decreasing the risk of developing metabolic and cardiovascular disorders (5).

A nationwide study was conducted in the United States from 2008 to 2014 to examine LPN versus RPN. It established that robotic approach has become a more favorable MIS option (23.9% vs. 92%) (6). Nevertheless, controversy exists in terms of outcomes, whether RPN or LPN is superior. Recent meta-analysis comparing both the techniques has indicated that RPN has more favorable outcomes in terms of conversion rates, effect on renal function, shorter hospital stay, and lower ischemic time (7).

In Saudi Arabia, studies regarding RCC and its management are rare. In the present study, we aimed to describe trends in PN management at our tertiary care referral center of King Abdullah International Medical Research Center, Riyadh, and compared the three approaches (RPN, LPN, and open partial nephrectomy [OPN]) in terms of outcomes, complications, and disease status on last follow-up as an initial local experience.

## Methods

Approval for the study was obtained from the Institutional Review Board (IRB) of King Abdullah International Medical Research Center. We did a retrospective chart review of all patients who had undergone PN between April 2013 and February 2019. At our tertiary care referral center, depending on factors pertaining to patient and surgeon’s experience and preference, PN is done either as OPN, LPN, or RPN using Da Vinci Si and Xi console robots. Patients were grouped according to the procedure type. Mostly, patients having clinical T1a and T1b renal masses undergo PN. Data variables collected included presentation, patient demographics (age, gender, body mass index [BMI], and size of tumor), RENAL nephrometry score (recorded by an experienced single reviewer), procedure type (OPN, LPN, or RPN), operative factors (operative time, ischemic time, estimated blood loss as declared by the surgeon at the end of the procedure, hospital stay, and perioperative blood transfusion), and complications; all these variables were classified using the Clavien grading system. Also, patients’ follow-up short-term (1–3 months) and long-term (6–12 months) data regarding hemoglobin, e*stimated glomerular filtration rate* (eGFR), creatinine, local recurrence, presence of metastasis, whether chemotherapy provided, and death were considered. Chi-square, ANOVA, and cross-tabulations were done to compare groups using SPSS software. P > 0.05 was considered significant.

## Results

In all, 69 patients, including 26 (37.7%) males and 43 (62.3%) females were identified, with the following parameters: mean age, 54.53 ± 13.21 years; mean BMI, 32.36 ± 7.03; and mean tumor size, 3.7 ± 1.72 cm (Table 1). In terms of presentation, most patients (N = 50, 72.4%) presented incidentally as opposed to symptomatic presentation with flank pain (14, 20.35%), hematuria (5, 7.2%), and dysuria (2, 2.9%) (Table 1). Most common comorbidity observed was hypertension (HTN) (34, 49.3%), followed by diabetes mellitus (DM) (30, 43.5%) and dyslipidemia (DLP) (20, 29%). Thirty-two (56.1%) patients had right-side tumor and 25 (43.9%) had tumor on their left side (Table 1); 18 patients (26.1%) underwent OPN, 29 (42%) LPN, and 22 (31.9%) RPN. Among these, 8 (11.6%) LPN procedures were converted to OPN; however, no RPN was converted to OPN (Table 1). Positive surgical margin score determined was 4 (22.2%) in OPN, 2 (6.9%) in LPN, and 7 (13%) in RPN; this difference was not statistically significant (P *=* 0.072), as the overall positive surgical margin rate was 13 (18.8%). No statistical significance was found between three PN modalities in terms of blood loss and operative time (P *=* 0.141 and 0.872, respectively). However, we found that OPN had more blood loss and a longer hospital stay but a shorter operating room (OR) time when compared with MIS PN, although this observation was not statistically significant (P *>* 0.05). Regarding hospital stay, we found that RPN had the least hospital stay between modalities, followed by OPN and LPN (P *=* 0.03) (Table 2). When comparing tumor complexity using the RENAL nephrometry score, we found that higher scores were used in OPN, followed by RPN and LPN; this was statistically significant (P *=* 0.02). In addition, ischemic time was longer in OPN, followed by LPN and RPN; this difference was also statistically significant (P *=* 0.014) (Table 2). The most common histological subtype of RCC found was clear cell in 41 patients (59.4%), followed by chromophobe in 10 (14.55), papillary in 6 (8.7%), oncocytoma in 4 (5.8%), and angiomyolipoma in 4 patients (5.8%). Regarding pathological grades, the most common, according to Fuhrman classification, was grade 2 in 33 patients (47.8%), followed by grade 3 in 10 (14.5%), grade 1 in 9 (13%), and grade 4 in 1 patient (1.4%). As for Clavien grading for complications is concerned, no statistically significant difference was found (P *>* 0.05) between modalities; however, it was observed that OPN had one grade-2 and one grade-4 complications as opposed to LPN, where only one patient had grade-1 complications, the rest had no complications. In the case of RPN, three patients had grade-1 and one had grade-2 complications, and the rest had no complications. No statistically significant difference was observed (P *=* 0.830) between pre-operative and day 1 post-operative hemoglobin values, which were measured for all patients, although it was observed that difference in mean hemoglobin values was highest in RPN at 19.72 mg/dL, followed by OPN at 18.22 mg/dL and LPN at 15.82 mg/dL. On follow-up, carried out at 6–12 months, we were able to measure estimated glomerular filtration rate (eGFR), creatinine, and blood urea nitrogen (BUN) levels; no statistically significant difference was found in eGFR and creatinine values between groups (P = 0.563 and 460, respectively). For BUN levels, statistical difference was observed between the groups (P *=* 0.024); the highest increase in BUN was observed in LPN group with a mean increase of 6.39 ± 21.70 mg/dL. Concerning patient status, we had one (1.4%) patient mortality, two patients (2.9%) had local recurrence, and one (1.4%) had lung metastasis, for which chemotherapy was commenced.

**Table 1:** Baseline characteristics of patients.

Variables		N (%)
Site	Right side	32 (56.1)
	Left side	25 (43.9)
Gender	Male	26 (37.7)
	Female	43 (62.3)
Procedure type	Open	18 (26.1)
	Laparoscopic	29 (42)
	Robotic	22 (31.9)
Complications	Converted to radical	1 (1.8)
	Converted to open	8 (11.6)
	Transfuse packed RBC	5 (7.2)
Presentation	Incidental	50 (72.4%)
	Hematuria	5 (7.2)
	Flank pain	14 (20.3)
	Dysuria	2 (2.9)
Comorbidities	HTN	34 (49.3)
	DM	30 (43.5)
	DLP	20 (29)
	CHF	1 (1.4)
	CAD	1 (1.4)
	Hypothyroid	4 (19.9)
Pathology	Clear cell	41 (59.4)
	Papilary	6 (8.7)
	Chromophobe	10 (14.5)
	Oncocytoma	4 (5.8)
	Angimyolipoma	4 (5.8)
Fuhrman grade	1	9 (13)
	2	33 (47.8)
	3	10 (14.5)
	4	1 (1.4)
On last follow-up	Mortality	1 (1.4)
	Lung metastasis	1 (1.4)
	Local recurrence	2 (2.9)
	On chemotherapy	1 (1.4)

HTN: hypertension; DM: diabetes mellitus; DLP: dyslipidemia; CHF: congestive heart failure; CAD: coronary artery disease.

**Table 2:** Baseline mean values of patients.

Mean values	
BMI mean *–*median ± SD	32.36–31.96 ± 7.03
Age mean (years)*–*median ± SD	54.53–53 ± 13.21
Hospital stay mean *–*median ± SD	6.88– 6 ± 4.939
Blood loss mean (mL)*–*median ± SD	297.16–150 ± 332.63
OR time (min.) mean *–*median ± SD	253.59–245 ± 63.66
Tumor size (cm) mean *–*median ± SD	3.7–3.5 ± 1.72
RENAL nephrometry score mean *–*median ± SD	6.23–7 ± 2.38
Ischemic time (min.) mean *–*median ± SD	22.32–20.05 ± 10.5

**Table 3:** Comparing groups for pre-operative and post-operative follow-up mean values for hemoglobin (Hgb), *glomerular filtration rate* (GFR), creatinine, and blood urea nitrogen (BUN), and mean values of operating room (OR) time, blood loss, hospital stay, tumor size, RENAL nephrometry score, and Ischemic time.

	Open	Laparoscopic	Robotic	P-value
Hgb mean difference ± SD post-op day 1	**18.22** ± 13.24	**15.82** ± 22.47	**19.72** ± 14.56	**0.830**
eGFR difference ± SD long-term 6 *–*12 months	**15.27** ± 15.27	**10.48** ± 35.06	**10.72** ± 32.80	**0.563**
Creatinine difference ± SD long-term 6 *–*12 months	**11.77** ± 44.30	**22.93** ± 41.56	**30.53** ± 31.78	**0.460**
BUN difference ± SD long-term 6 *–*12 months	**1.30** ± 2.22	**6.39** ± 21.70	**1.95** ± 2.77	**0.024**
OR time (min) ± SD	**225.39** ± 55.93	**271.29** ± 63.12	**254.14** ± 66.07	**0.872**
Blood loss (mL) ± SD	**542.22** ± 720.67	**334.14** ± 564.01	**245.45** ± 212.04	**0.141**
Hospital days ± SD	**7.61** ± 5.00	**7.62** ± 6.22	**5.32** ± 1.83	**0.031**
Tumor size (cm) ± SD	**4.23** ± 1.72	**3.56** ± 1.93	**3.45** ± 1.48	**0.973**
RENAL nephrometry score ± SD	**7.22** ± 1.39	**5.41** ± 2.89	**6.50** ± 1.94	**0.020**
Ischemia time (min) ± SD	**27.87** ± 11.51	**22.93** ± 13.13	**19** ± 4.89	**0.014**

## Discussion

For managing small renal masses, PN has replaced radical resection as the standard of care (3, 4). PN offers an excellent oncological control of RCC and is popular among urologists because this nephron sparing procedure entails higher survival rates and is less morbid than radical resection. With increased incidental diagnoses, PN has proved to be an acceptable approach for small RCC (8, 9). Nevertheless, PN carries the risk of local disease recurrence, which has been reported in as high as 10% patients in a larger series (10). This has come down with the development of RPN, and according to a recent meta-analysis, RPN has lowered the incidence of positive surgical margins as well as have more favorable perioperative results, thus enabling to effectively manage complex tumors in an MIS setting (7). Lack of studies addressing the management of small renal masses with different modalities in Saudi Arabia has encouraged us to report our local experiences.

In this study, we demonstrated the initial Saudi experience of managing RCC in 69 patients treated with PN at tertiary care center, and compared OPN, LPN, and RPN. Although larger number series and long follow-up outcomes were required, PN offered excellent oncological control of small RCC tumors, and with the use of MIS, it offered favorable outcomes. In our patient population, the overall positive surgical margin rate was 18.8%, which was higher than what most series have reported (0–10%) irrespective of PN modality (11, 12). The impact of having a positive surgical margin has been reported by some larger series, with insignificant or no impact on metastasis or recurrence (13). High positive margin rate in our study population may be attributed to factors such as large mean tumor size, which was less than 4 cm with a mean size of 3.7 cm. Yossepowitch et al. (14) have reported that a larger tumor size is associated with lower positive margin rates in univariate and multivariate analyses. Positive surgical margin rate is affected by factors such as surgeon experience, use of intraoperative ultrasound, frozen section, and wide excision if margin integrity is questioned interop (15).

In our study population, no effect was observed on renal function between all groups except elevated BUN in LPN group. Nonetheless, all the observed parameters across three groups didn’t show any clinically significant decline in renal function on follow-up, which was >25% decline in GFR from baseline according to recommendations and guidelines (13). However, to accurately measure the effect of PN on renal function, renal arterial resistive index measurement is required through duplex ultrasonography (16).

Our study is distinctive in its finding that PN outcomes are comparable across all modalities. This is especially remarkable when looking at LPN, as this approach is known to be technically challenging, has a lengthy learning curve to master, and was the most common approach used in our study (17). On the other hand, RPN is relatively new in Saudi Arabia and not many centers have access to a surgical robot, although this choice, according to prospective studies, has proven to be a viable, safer, and more effective option (18). Hopefully, with more RPN cases and introduction of surgical robots at more centers, results may be achieved comparable to high-volume centers across the world.

We compared all three modalities of PN in terms on ischemic time and RENAL nephrometry score. According to a recent systematic review, ischemic time during PN has no effect on renal function unless it is longer than the limit of 25 min (19). In our study, between three modalities of PN, we established that OPN had the longest ischemic time, followed by LPN and RPN. This finding contradicts the US retrospective study comparing all three modalities of PN for ischemic time, where they had reported a shorter ischemic time in OPN, followed by RPN and LPN. The complexity of renal tumor, demonstrated by high RENAL nephrometry score in OPN patients, may partially explain longer ischemic time (20). RENAL nephrometry score, which has proven to be a reliable tool, was developed to assess tumor complexity (21). In our study, the mean RENAL nephrometry score was 6.2, with higher scores observed in OPN (7.22) and RPN (6.5) but the lowest in LPN (5.41); this difference in RENAL nephrometry score was statistically significant (P *=* 0.02). This was reported by a multicenter study conducted in the United States. This US study examined 2392 MIS PN cases over a period of 9 years and found that RPN had significantly higher RENAL nephrometry score compared to LPN (P *<* 0.001) (22).

## Limitations

Our study is not without limitations. The retrospective nature of the study is limited due to its design. In addition, its relatively small sample size and its setting in tertiary care referral center may not yield a truly representative sample of the Saudi population. Furthermore, surgeons from different backgrounds with varying training and operating experiences performed these procedures. This has been shown in recent reports to affect all aspects and outcomes of surgical management of RCC with PN, except its effect on renal function, which corresponds with the results of our study (23). In addition, our study did not measure success of PN using the concepts of trifecta and pentafecta; these are the most common methods for assessing both short- and long-term PN success. This was mostly due to missing data and loss of follow-up in our study population (24).

## Conclusion

This is an initial local study addressing the management of RCC in Saudi Arabia. Our study establishes that PN outcomes between OPN, LPN, and RPN are similar if conducted by experienced surgeons. However, open procedures have higher blood loss, higher OR time, and longer hospital stay when compared with minimally invasive techniques. With our modest experience, we have demonstrated that PN is a feasible option in all its approaches, but more experience is required to achieve results similar to that of high-volume centers.
